# Influence of Clinical and Genetic Factors on the Progression of Age-Related Macular Degeneration: A 3-Year Follow-Up

**DOI:** 10.3390/jcm12051963

**Published:** 2023-03-01

**Authors:** Elżbieta Krytkowska, Zofia Ulańczyk, Aleksandra Grabowicz, Krzysztof Safranow, Miłosz Piotr Kawa, Andrzej Pałucha, Anna Wąsowska, Ewa Matczyńska, Anna Boguszewska-Chachulska, Anna Machalińska

**Affiliations:** 1 First Department of Ophthalmology, Pomeranian Medical University, 70-111 Szczecin, Poland; 2 Department of General Pathology, Pomeranian Medical University, 70-111 Szczecin, Poland; 3 Department of Biochemistry and Medical Chemistry, Pomeranian Medical University, 70-111 Szczecin, Poland; 4 Genomed SA, 02-971 Warsaw, Poland; 5 Department of Ophthalmology, Faculty of Medical Sciences in Zabrze, Medical University of Silesia, 40-760 Katowice, Poland

**Keywords:** age-related macular degeneration (AMD), progression, CFH Y402H, ARMS2 A69S, PRPH2, choroid, thyroid hormones

## Abstract

The aim of the present study was to analyze the relationship of age-related macular degeneration (AMD) progression with clinical characteristics, demographic, and environmental risk factors that would affect disease development. In addition, the influence of three genetic AMD polymorphisms (CFH Y402H, ARMS2 A69S, and PRPH2 c.582-67T>A) on AMD progression was investigated. In total, 94 participants with previously diagnosed early or intermediate AMD in at least one eye were recalled for an updated re-evaluation after 3 years. The initial visual outcomes, medical history, retinal imaging data, and choroidal imaging data were collected to characterize the AMD disease status. Among the AMD patients, 48 demonstrated AMD progression, and 46 showed no disease worsening at 3 years. Disease progression was significantly associated with worse initial visual acuity (OR = 6.74, 95% CI = 1.24-36.79, *p* = 0.03) and the presence of the wet AMD subtype in fellow eyes (OR = 3.79, 95%CI = 0.94-15.2, *p* = 0.05). In addition, a higher risk of AMD progression appeared in the patients with active thyroxine supplementation (OR = 4.77, CI = 1.25–18.25, *p* = 0.002). The CC variant of CFH Y402H was associated with AMD advancement compared to the TC+TT phenotype (OR = 2.76, 95% CI: 0.98–7.79, *p* = 0.05). Identifying risk factors of AMD progression may lead to earlier intervention and better outcomes, preventing the expansion of the late stage of the disease.

## 1. Introduction

Macular degeneration is a known major cause of vision loss in people over 60 years of age, and its frequency increases with age [1]. The number of people living with age-related macular degeneration (AMD) was expected to reach 196 million worldwide in 2020 and is estimated to increase to 288 million by 2040 [2]. In developed countries, approximately 10% of the population over the age of 65 years and 25% over the age of 75 years have been diagnosed with different forms of AMD. It is further estimated that 85% of all AMD patients have early and intermediate AMD, while 15% of all AMD patients are affected by the advanced stages of the disease, including central geographic atrophy, active choroidal neovascularization, or inactive choroidal neovascularization [3]. Importantly, no treatment options are available for the early and intermediate stages of AMD. Intravitreal anti-VEGF therapy for wet AMD is also unsatisfactory because 25% of eyes achieve visual acuity of 0.1 or less within 5 years despite active treatment [4]. The lack of effective treatment is due, in part, to the complexity of the disease as not only multiple genetic loci but also environmental risk factors have been shown to be involved in AMD pathology. During the last several decades, different large observational cohort- and population-based research studies have documented strong evidence supporting associations between AMD development and diverse risk factors, such as sociodemographic features, medical status, and selected biochemical variables [5,6]. However, there are inconsistencies among the literature data regarding the factors that may affect AMD progression. Therefore, we performed an analysis of demographic data, lifestyle data, medical history, and existing comorbidities combined with genetic risk factors for AMD and evaluated the relevance of such features for AMD progression over 3 years. All subjects who participated in this follow-up were submitted to multimodal retinal imaging and underwent a general medical questionnaire. The aim of the present study was to examine the relationship of AMD progression with clinical characteristics, demographic, and environmental risk factors that would affect disease development over a period of time. Furthermore, in 2018, our group were the first to report the relationship between the presence of the peripherin gene (PPH2) c.582-67T>A variant and the occurrence of AMD [7]. The peripherin gene (PRPH2) encodes specific proteins that are crucial for the proper formation and function of the outer segments of rods and cones [8]. Thus, we investigated the influence of three genetic AMD polymorphisms (CFH Y402H, ARMS2 A69S, and PRPH2 c.582-67T>A) and their effect on AMD deterioration.

## 2. Materials and Methods

### 2.1. Subjects and Initial Management

Initially, 244 patients with clinically diagnosed early or intermediate AMD in at least one eye were randomly selected from among the patients of the outpatient clinic of the 1st Ophthalmology Department in 2016–2017. The follow-up examination was planned for 2019–2020. Due to the COVID-19 pandemic and the massive disruption to healthcare, 150 patients did not turn up for a follow-up checkup within 3 years or refused to participate in the project altogether. Finally, 94 people were followed up, which was 38.5% of the originally recruited participants. Data on medical history, current drug use, smoking status, and physical activity from the enrolled subjects, with a particular focus on their history of clinical cardiovascular diseases, including physician-diagnosed heart and vascular diseases, were collected. We also assessed waist circumference [cm], waist/hip ratio (WHR), and body mass index (BMI) [weight (kg)/height (m)^2^] of all enrolled subjects. The reported average number of cigarettes smoked per day and the number of years the patient smoked were collected to calculate cumulative pack-years. Finally, with the help of a member of the research team, each participant completed the International Physical Activity Questionnaire (IPAQ), comprising seven questions regarding all types of physical activity (lasting 10 min or longer) in the previous week. Physical activity scores are presented as MET-min per week and were calculated as described previously [9]. Medical history was taken personally by a medical worker (doctor or trained nurse), on the basis of the data provided by patients and their medical records, including a statement from a primary care physician. In accordance with the tenets of the Declaration of Helsinki, a consent form was signed by all patients before trial enrolment.

### 2.2. Ophthalmologic Examination

The patients underwent a detailed ophthalmologic examination as follows: measurement of visual acuity using the logarithm of the minimum angle of resolution (LogMAR) charts; slit-lamp examination of the anterior and posterior eye segments; color fundus photography (CFP); and Goldmann applanation tonometry. Axial length and anterior chamber depth were assessed to eliminate known factors that interfere with reliable OCT image analysis. Additionally, fundus autofluorescence (FAF) and near-infrared reflectance (NIR) imaging examinations were performed for more accurate drusen visualization to allow correct determination of the AMD stage. In eyes where the presence of CNV could not be unequivocally excluded, fluorescence angiography was performed. Patients presenting with ocular media opacity due to corneal, lens, or vitreous changes potentially influencing OCT scan quality were excluded. The exclusion criteria also encompassed ophthalmic and systemic conditions that may affect the choroidal thickness, such as high refractive errors (over ±4 diopters of spherical equivalent), glaucoma, choroiditis, retinopathy (any type), vitreomacular changes (such as an epiretinal membrane), a history of retinal detachment, or serious ocular trauma. Eyes were excluded if they had cataract surgery in the previous 6 months, as well as any previous laser therapy or vitreoretinal surgery.

To obtain EDI-OCT images of the macular region, a 30° ×  25° volume acquisition protocol was used, achieving 61 cross-sectional B-scans (Spectralis, Heidelberg Engineering, Germany). The measurements were performed by an experienced technician after pupil dilation with a 1% tropicamide solution after 30 min of rest and at the same time of the day (between 9.00 and 11.00 a.m.). FAF images were obtained using an excitation blue light of 488 nm and a barrier filter beginning at 500 nm. High-contrast digital NIR images were acquired using the 815 nm diode laser of the Spectralis System. The images were captured at the time of acquisition of the macular OCT scans.

Grading of AMD based on the evaluation of color fundus and OCT images using the Ferris classification system was performed by the examining clinician [10]. The early AMD group included patients with medium drusen (63–125 μm) but without pigmentary abnormalities. The intermediate AMD group comprised patients who had large drusen or pigmentary abnormalities associated with at least medium drusen. Patients with lesions associated with neovascular AMD or geographic atrophy were classified as having late AMD. Geographic atrophy was defined by the presence of sharply demarcated atrophic lesions of the outer retina, resulting from loss of photoreceptors, RPE, and underlying choriocapillaris layer based on CFP, FAF, and NIR imaging [11]. Wet form AMD was diagnosed in eyes with features of pathological vessels that were confirmed by ophthalmoscopy, coherence tomography, or fluorescein angiography.

We analyzed the influence of the size and type of drusen on the risk of AMD progression. The largest drusen in the circle area with a diameter of 6 mm centered in the fovea was manually evaluated. The size of the drusen was estimated as the product of their height (the distance between Bruch’s membrane and the RPE at the highest point of the druse) and their width at the base along the BrM. The type of drusen was determined based on its appearance in color fundus photography, OCT, and FAF images. Hard drusen were described as punctate, small-medium sized (<125 μm), and yellow–white lesions with discrete borders observed in CFP, OCT, and IR images. Soft drusen were identified as yellow–white mound-like elevations with blurred boundaries and gradually reduced density from the center to the periphery observed in CFP images, which corresponded to the moderately reflective dome-shaped sub-RPE deposits present on OCT scans [12,13]. Pachydrusen were identified when isolated or scattered yellow–white deposits, which had a better-defined and more complex outer border compared to regular round soft drusen, were observed in CFP images [14]. On the OCT scans, pachydrusen were located adjacent to the pachyvessels [15]. Subretinal drusenoid deposits (SDD) were recognized when discrete white–yellowish interlacing network deposits were present in the color photography, and they corresponded with a round or triangular, well-defined, hyper-reflective subretinal deposit accumulation of material that forms sharp peaks and may lead to abruption of the inner and outer photoreceptor segment layer on the OCT scans. On FAF and IR images, SDD were observed as an area of hyperautofluorescence/reflectance on the center of each reticular pseudodrusen surrounded by a hyporeflective halo, forming the characteristic appearance of the target [16,17].

The diagnosis of pachychoroid was based on the presence of subfoveal choroidal thickness >350 μm or an extrafoveal focus that exceeded fovea choroidal thickness by at least 50 μm as well as the presence of pachyvessels identified on EDI-OCT b-scans [18,19,20]. Pachyvessels were defined as dilated outer choroidal vessels compressing the overlying choriocapillaris and Sattler’s layers observed on cross-section EDI-OCT images [20,21,22]. Only good quality scans with well-defined choroid-scleral junction were further analyzed.

To estimate disease progression, all abovementioned ophthalmologic tests were repeated after 36 months. To identify the impact of specific systemic and ocular parameters on AMD deterioration, patients were assigned to one of two subgroups depending on the status of disease progression. AMD progression was defined as the change in disease stage from early to intermediate AMD as well as the presence of late-stage disease features in the eyes previously classified as early or intermediate in at least one eye. No AMD progression was established when none of the signs of a more advanced stage of disease were observed.

### 2.3. Genotyping

Venous blood samples (approximately 7.5 mL) collected in EDTA tubes were centrifuged (2000 rpm, 4 °C, 10 min), and red blood cells were lysed using ammonium chloride-based lysing buffer (BD Biosciences, Franklin Lakes, NJ, USA). Nucleated cells were then counted, and DNA isolation was subsequently performed with a total DNA isolation kit (Macherey-Nagel, Düren, Germany) according to the manufacturer’s protocol. AMD-risk polymorphisms were genotyped as previously described [7]. In CFH, rs1061170, encoding a Y402H interchange, was genotyped by restriction analysis with EagI, HhaI, and Hsp92II enzymes. In ARMS2, LOC387715 rs10490924, encoding an A69S interchange, was determined by direct DNA sequence analysis using an Applied Biosystems 3130 XL instrument for DNA sequencing. The genetic analysis of the PRPH2 gene was performed by exon capturing with Molecular Inversion Probes (MIPs) and subsequent sequencing of amplified libraries with the Illumina NextSeq 500 system [7]. Molecular analysis was performed by the Genomed SA bioinformatic team as well as an outwards cooperating laboratory according to generally approved standards.

### 2.4. Statistical Analysis

Qualitative variables were compared between groups with Chi-squared or Fisher’s exact tests. Because the distributions of most quantitative variables were significantly different from a normal distribution, the nonparametric Mann–Whitney test was used for their analysis. A logistic regression model adjusted for age and sex was used for multivariate analysis of potential predictors of AMD progression. Odds ratio (OR) and 95% confidence interval (95% CI) values were calculated for comparison of the AMD progression group to the no progression group, which was considered a reference. OR values for quantitative parameters reflected odds of AMD progression associated with an increase in a parameter by one unit (continuous variables) or class (rank variables). *p* < 0.05 was considered statistically significant.

## 3. Results

The follow-up data (36 months after the initial evaluation) were collected from 94 patients. Among the study subjects, 48 patients presented with AMD progression, and 46 patients showed no disease worsening.

### 3.1. Systemic Factors Associated with AMD Progression

We aimed to assess the effects of systemic factors on AMD progression. The data regarding demographic factors, medical history of the patients, and medication use are presented in Table 1. Patient age, sex, iris color, and AMD family history were not associated with disease advancement. There was also no association between the presence of selected, well-defined systemic diseases (such as hypertension, ischemic heart disease, cardiac infarction, peripheral artery disease, or limb ischemia) and AMD progression. Similarly, there was no association between medication use, except for thyroxine use, and disease development. Thyroxine use was a significant predictor of disease deterioration (OR = 3.75, 95% CI = 1.08–12.97, *p* = 0.05), and this association remained significant in multivariable logistic regression analysis adjusted for age, sex, and presence of ischemic heart disease (OR = 4.77; 95% CI = 1.25–18.25, *p* = 0.002).

### 3.2. Clinical Parameters Associated with AMD Progression

We next discriminated the initial clinical features of the disease that were linked with AMD advancement, e.g., visual acuity, choroidal thickness, retinal thickness, drusen type, drusen size, AMD stage, and wet AMD subtype in one eye. Patients with worse initial visual acuity scores were more likely to develop AMD progression (OR = 3.56, 95% CI = 0.96–13.1, *p* = 0.04) after 3 years of observation. This association remained significant in multivariate logistic regression analysis adjusted for age and sex (OR = 6.74, 95% CI = 1.24–36.79, *p* = 0.03). Interestingly, in patients who initially presented with thinner choroids, AMD advancement was more likely in the follow-up (OR = 0.99, 95% CI = 0.99–1.00, *p* = 0.049). However, in a multivariate regression analysis by sex and age, this factor was not significantly associated with AMD progression (OR = 0.1, 95% CI = 0.99–1.0, *p* = 0.12). Neither the presence of pachychoroid features nor the coexistence of pachyvessels in patients with AMD were significantly associated with disease progression.

Among the clinical parameters, the wet AMD subtype in one eye was a particularly important predictor of disease deterioration in the other eye (OR = 4.17, 95% CI = 1.06–16.46, *p* = 0.04), and this association remained significant in multivariate analysis adjusted for age and sex (OR = 3.79, 95% CI = 0.94–15.2, *p* = 0.05). There was no association of disease progression with initial retinal thickness, drusen size, and type or AMD stage. Detailed results are presented in Table 2 and Table 3.

Next, to test whether patients with AMD who progressed to late stage differ from those who progressed to intermediate stage, we performed a comparative analysis of the two subgroups. The subgroup that progressed to the late stage consisted of 35 patients, while the subgroup that progressed to the intermediate stage consisted of 13 participants. There were no significant differences between the groups in terms of age, gender distribution, and the coexistence of systemic vascular diseases and their risk factors. Accordingly, the analysis showed no significant differences in thyroxine supplementation (*p* = 0.73), initial visual acuity (*p* = 0.34), and central choroidal thickness (*p* = 0.11) between the groups. Detailed results are available in Tables S2–S4 in Supplementary Materials.

### 3.3. Genetic Factors Influencing AMD Progression

Finally, we analyzed whether the genetic background of the patients influences AMD progression (Table 3). We selected three single nucleotide polymorphisms (SNPs) for this analysis, namely, CFH Y402H, ARMS2 A69S, and PRPH2 c.582-67T>A, which were strong AMD risk factors in our previous study [7]

In multivariable logistic regression analysis adjusted for age, sex, and initial visual acuity, the CC variant of CFH Y402H was significantly associated with AMD advancement compared to the TC + TT phenotype (OR = 2.76, 95% CI: 0.97–7.79, *p* = 0.05). The tested variants of the ARMS2 and PPH2 genes did not show a significant relationship with the progression of AMD. Detailed results are presented in Table 3 and Table 4.

## 4. Discussion

In our cohort, the multivariate logistic regression analyses failed to identify any significant differences in the current and past medical status or demographic characteristics between those who presented with disease progression and those who showed no AMD worsening. Similarly, we found no association between medication use, except for thyroxine use, and disease advancement. Thyroxine use was a significant predictor of AMD progression. Thyroid dysfunction (both hypo- and hyperthyroidism) has been previously analyzed in the context of an increased risk of AMD [23]. Interestingly, a positive association between levels of fT4 and macular degeneration was observed in the prospective, population-based Rotterdam study performed on approximately 15,000 subjects recruited at different ages and observed over more than two decades [24]. Importantly, even in restricting analyses to euthyroid individuals with a normal range of fT4, higher fT4 values were still associated with an increased risk of AMD development in the study. Furthermore, Farvadin et al. showed a relationship between serum free T4 levels and wet AMD. [25] Gopinath et al., in multivariate regression analysis adjusted for age, sex, current smoking, fish consumption, and presence of high risk CFH and ARMS2 variants, demonstrated that overt hyperthyroidism (low TSH and high free T4 levels) in older patients is independently associated with a 3-fold increase in the risk of AMD development; however, they did not verify any significant positive association between serum free T4 levels and incident AMD in their study. Researchers have also found an association between the development of AMD and thyroxine supplementation in both current and past users [26]. The Beaver Dam Eye study also reported an association between therapeutic thyroid hormone use and early AMD, while a case-control study of the Age-Related Eye Disease Study Research Group reported an association between thyroxine supplementation and a higher risk of geographic atrophy [27,28]. However, there is no consensus among researchers regarding the association between thyroid medications and the risk of AMD. While the meta-analysis by Xu et al. published in 2021 confirmed that thyroid disorders may increase the risk of AMD, no link to the use of thyroid medications was found [29]. Thyroid hormone signaling regulates retinal development and maintenance via regulating cone maturation and expression of cone opsins [30]. Hyperthyroidism has been observed to accelerate the basal metabolic rate and oxidative metabolism by inducing mitochondrial enzymes, which may induce local cellular oxidative stress [31]. Recent molecular and in vivo studies have implicated thyroid hormone signaling in cone photoreceptor viability [32]. It has been discovered that inhibition of thyroid hormone signaling protects photoreceptors from oxidative stress-induced cell death in a mouse model of age-related macular degeneration [33]. However, there is evidence that thyroid hormones may negatively influence retinal pigment epithelium (RPE) cells, resulting in further degeneration of photoreceptors [34]. Furthermore, it has been observed that deficiency of type 2 iodothyronine deiodinase, which is the crucial enzyme for fT4 cellular metabolism, reduces cellular necroptosis and the activity of oxidative stress responses in retinas in a mouse model of Leber congenital amaurosis [35]. These findings may serve as molecular evidence for the causal inference between macular/retinal degeneration and increased concentrations of thyroid hormones due to natural or pharmacological reasons, implicating the specific pathophysiologic role of these hormones in the progression of AMD. Importantly, thyroid dysfunction is related to vascular function, atherosclerosis, and cardiovascular disease [36]. Both oxidative stress and vascular dysfunction make choroid and retinal tissue more susceptible to the development of degenerative changes. Taken together, these findings warrant the need for further research to investigate the biological relationship between AMD progression and thyroid hormone imbalance, especially because it is a modifiable risk factor. We did not find the AMD progression to be related with baseline clinical characteristics such as retinal and choroidal thickness, drusen size, and drusen type. However, we observed that worse initial visual acuity and wet AMD subtype in one eye significantly correlated with AMD advancement. Progression from early to late AMD is associated with severe and irreversible vision loss [37]. Therefore, deteriorating vision and its decreased acuity are important outcome measures for defining AMD progression in prospective clinical trials [38]. In a group of more than 40,000 patients from Great Britain with a diagnosis of early/intermediate AMD, Chakravarthy et al. observed that better VA was significantly associated with a decreased rate of progression to advanced AMD [39]. Studies of wet AMD (wAMD) eyes treated with AVEGF have shown that patients with good initial visual acuity are more likely to maintain good vision during 2 years of follow-up [40,41]. Some researchers have emphasized the relationship between VA and choroidal thickness in both healthy and AMD eyes. [42,43,44] Baseline CT is also regarded as a predictive factor of VA outcome in patients with wAMD. In a case series study, greater initial subfoveal choroidal thickness was found to be associated with a better anatomic and functional clinical outcome in eyes with wAMD after intravitreal aflibercept therapy [45]. Eyes with an initially thinner choroid thickness may have a lower “choroid reserve.” In the age-related decrease in choroidal flow, impaired nutrition of the RPE, as a result of insufficient blood supply to the choroid, leading to progressive loss of photoreceptors and deterioration of visual acuity, may appear earlier or be more pronounced compared to eyes with higher CT. However, some studies have reported no correlation between choroidal thinning and worsening retinal function in AMD eyes [46]. In the current study, subfoveal choroidal thickness was not risk a factor of AMD progression in multivariate regression analysis. Several studies have investigated whether decreased choroidal thickness is a risk factor for progression to advanced AMD. Lee et al. reported that a reduced subfoveal CT correlates with a higher progression rate of nonexudative AMD. [43] In another study, researchers have identified that a thinner choroid is a risk factor for the development of new onset atrophy in dry AMD eyes with drusenoid lesions. [47] Similarly, Fan et al. reported that AMD patients with decreased subfoveal CT at baseline are more likely to develop advanced macular atrophy at the 18-month follow-up [48]. Another prospective study has reported that irregular choroidal vessels are predictive of earlier development of both late disease forms, namely, GA and neovascular AMD [49]. However, not all studies clearly indicate a relationship between AMD progression and the presence of a thinner choroid. In the study, Manjunath et al. showed that patients with AMD have a wide range of choroidal thicknesses in different stages and subtypes of the disease [50]. Similarly, Keenan et al. showed that in AMD, the choroid undergoes bidirectional changes as it becomes thicker as drusen develops in the early stages of the disease and thinner in the later stages [51]. It seems that the most valuable studies on the role of the choroid in AMD will be those based on long-term observation of the patient in all phases of the disease and analyses, taking into account local and systemic confounding factors.

Interestingly, Sakurada et al. reported that the unilateral neovascular AMD subtype is a particularly important predictor of disease deterioration in the other eye during the mean follow-up period of 36 months [52]. Gangnon et al. stated that severe AMD in the study eye is associated with an increased incidence of AMD and accelerated progression in its fellow eye [53]. Similarly, the prospective, longitudinal study by Silva et al. reported a 3-year progression from early to late exudative AMD in fellow eyes of patients with wet AMD in the other eye [54]. Moreover, patients with early/intermediate AMD and a diagnosis of CNV in the fellow eye have the fastest rate of disease progression. [45,55] These findings strongly support the results obtained from the present study.

It has been confirmed that AMD has a strong genetic basis. To date, more than half of the genomic heritability of AMD can be explained by genetic variants in 34 known AMD-associated loci, which are mostly involved in the regulation of complement system activation [55]. Common SNPs within CFH, including rs1061170 that encodes a tyrosine to histidine substitution at position 402 (Y402H), were first identified as major susceptibility variants for AMD [56]. Multiple prospective studies have confirmed that the minor allele of this variant is a risk allele for the incidence of early AMD (HR 1.2–2.3) as well as for the progression to both late forms of AMD, such as wet AMD (HR 1.48–2.5) and geographic atrophy (HR 1.38–3.65) [57,58,59]. In the present study, the multivariable logistic regression analysis adjusted for age, sex, and visual acuity revealed that among the tested AMD-associated loci, the CC variant of CFH Y402H was associated with AMD advancement over a 3-year period with borderline significance. This finding confirmed data from the newest meta-analyses based on pooled studies, including 17,174 individuals 45 years of age or older participating in 6 population-based cohort studies, 2 clinic-based studies and 1 case‐control study, demonstrating that CFH risk variants are the leading drivers of late forms of AMD [60,61]. In contrast, the PRPH2 variant c.582-67T>A (rs3818086) showed no association with AMD progression. This may indicate that this gene plays a role in the pathomechanism associated with the onset of the disease, but not in its progression, which requires further research in a larger group of patients.

## 5. Conclusions

In conclusion, the 3-year follow-up of AMD patients recruited to a single-center, prospective study confirmed that worse visual acuity as well as the presence of unilateral CNV are a strong risk factor for AMD progression. The presence of this feature implies particular vigilance in patient management and in planning follow-up visits. Identifying modifiable risk factors for disease progression, such as thyroid hormone imbalance, is particularly valuable as a potential target of prevention and requires further study in a larger and more homogeneous group of patients.

## Figures and Tables

**Table 1:** Characteristics of the study subjects. Disease progression with regard to demographic data and medical history. The data are presented as the mean ± SD or %.

Parameter		AMD Progression	No AMD Progression	OR (95% CI)	*p*-Value *
Number of subjects		48	46	—	—
Sex (male/female)		16/32	16/30	0.93 (0.39–2.22)	1.00
Patient’s age [years] (min–max)		72.7 (56–84)	70.4 (54–85)	1.04 (0.98–1.11)	0.07
Iris colour (dark/light)		11/37	18/28	0.46 (0.19–1.15)	0.12
Education	Basic (%)	50%	50%	1.03 (0.66–1.60)	0.99
	Vocational (%)	50%	50%		
	Secondary (%)	52.5%	47.5%		
	Higher (%)	51.7%	48.2%		
AMD family history		46.7%	53.3%	0.81 (0.26–2.49)	0.78
Currently smoking		36.4%	52.6%	0.51 (0.14–1.94)	0.35
Formerly smoking		41.9%	58.7%	0.51 (0.21–1.19)	0.14
BMI (kg/m^2^)		26.8 ± 4.2	27.0 ± 4.9		0.68
Physical activity (MET)		1624.5 ± 2079.3	1890.4 ± 2501.8		0.24
Medical history					
Hypertension		50%	50%	0.93 (0.38–2.30)	1.00
History of ischemic heart disease		55.6%	50.6%	1.22 (0.30–4.98)	1.00
History of myocardial infarction		60%	50,6%	1.46 (0.23–9.47)	1.00
History of peripheral artery disease		60%	50.6%	1.46 (0.23–9.47)	1.00
History of limb ischemia		60%	50.6%	1.46 (0.23–9.47)	1.00
Medications use					
Hypotensive drugs/vasodilators		50.8%	50%	1.03 (0.41–2.57)	1.00
Thyroxine		75%	44.4%	3.75 (1.08–12.97)	**0.05**
Steroids		50%	50%	1.00 (0.06–17.18)	1.00
Statins		48%	52%	0.87 (0.34–2.22)	0.82
NSAIDs		55.6%	49.3%	1.28 (0.45–3.69)	0.79
Cardiac medications/antiarrhythmic drugs		50%	50.6%	0.45 (0.10–1.97)	0.31
Antiasthmatic drugs		75%	49.4%	3.07 (0.30–31.74)	0.62
Antidepressants		75%	49.4%	3.07 (0.30–31.74)	0.62
Vitamins and antioxidants		50%	51.6%	0.94 (0.39–2.27)	1.00
Xanthines (lutein, zeaxanthin)		53.3%	44.8%	1.41 (0.57–3.47)	0.50
Omega-3 rich oils		51.9%	48.7%	1.14 (0.48–2.68)	0.83
Resveratrol		54.8%	46.81%	1.37 (0.59–3.21)	0.53

* Mann–Whitney/Chi-squared or Fisher’s exact test. The statistically significant result is marked in bold.

**Table 2:** State of disease progression with regard to the baseline clinical characteristics of the study subjects. The data are presented as the mean ± SD.

Clinical Parameter		AMD Progression	No AMD Progression	OR (95% CI)	*p*-Value *
Visual acuity (logMAR)		0.49 ± 0.35	0.35 ± 0.31	3.56 (0.96–13.10)	**0.04**
Choroidal thickness in the foveal region (μm)		207.5 ± 84.3	245.4 ± 96.6	0.995 (0.990–1.00)	**0.0497**
Pachychoroid (Y/N)		4/44	1/44	4.00 (0.42–38.37)	0.36
Pachyvessels (Y/N)		19/29	19/26	0.90 (0.38–2.07)	0.83
Retinal thickness in the central ETDRS area (μm)		317.4 ± 80.8	300.6 ± 55.0	1.00 (1.00–1.00)	0.52
Drusen size		2.43 ± 0.65	2.43 ± 0.73	0.99 (0.54–1.81)	0.80
Soft drusen		65%	69%	0.82 (0.34–1.98)	0.83
Hard drusen		25%	22%	1.17 (0.44–3.08)	0.81
Subretinal drusenoid deposits (SDD)		42%	24%	2.21 (0.90–5.44)	0.12
Pachydrusen		10%	11%	0.93 (0.25–3.52)	1.00
AMD stage	Early	17.0%	8.7%	1.03 (0.56–1.90)	0.18
	Intermediate	38.3%	56.5%		
	Late	44.7%	34.8%		
Wet AMD in one eye (yes [%])		37.5%	32.6%	4.17 (1.06–16.46)	**0.04**

* Mann–Whitney/Fisher’s exact test. Statistically significant results are marked in bold.

**Table 3:** Multivariate logistic regression analysis of patients considering the presence of AMD progression as a dependent variable, adjusted for age and sex.

Independent Variables	OR	−95% CI	+95% CI	*p*-Value
Thyroxine supplementation	6.42	1.6	25.79	**0.008**
Visual acuity (logMAR)	5.1	1.05	24.69	**0.039**
Choroidal thickness in the foveal region (μm)	0.1	0.99	1.0	0.12
Wet AMD in one eye (yes [%])	0.99	4.49	20.3	**0.048**
CFH	2.76	0.98	7.79	**0.05**

Statistically significant results are marked in bold.

**Table 4:** State of disease progression with regard to the genetic background of patients.

Tested SNP	Genotype	% of Patients with AMD Progression	% of Patients without AMD Progression	*p*-Value *	Genotypes or Alleles	OR (95% CI)	*p*-Value *
*CFH* Y402H	TT	41.2%	58.8%	0.19	CC + TC vs. TT	1.47 (0.50–4.36)	0.48
					CC vs. TC + TT	2.40 (0.92–6.21)	0.069
	TC	41.7%	58.3%		CC vs. TT	2.43 (0.70–8.41)	0.16
					C vs. T allele	1.69 (0.90–3.18)	0.10
	CC	63.0%	37.0%		TC vs. TT	1.02 (0.32–3.29)	0.97
					CC vs. TC	2.38 (0.85–6.63)	0.09
*ARMS2* A69S	GG	46.9%	53.1%	0.69	TT + GT vs. GG	1.09 (0.44–2.65)	0.85
					TT vs. GT + GG	1.91 (0.42–8.60)	0.39
	GT	46.3%	53.7%		TT vs. GG	1.89 (0.38–9.27)	0.43
					T vs. G allele	1.18 (0.62–2.26)	0.61
	TT	62.5%	37.5%		GT vs. GG	0.98 (0.39–2.47)	0.96
					TT vs. GT	1.93 (0.41–9.16)	0.40
*PRPH2* c.582-67T>A (rs3818086)	TT	42.1%	57.9%	0.13	AA + TA vs. TT	1.37 (0.49–3.88)	0.55
					AA vs. TA + TT	0.40 (0.14–1.20)	0.098
	TA	58.1%	41.9%		AA vs. TT	0.63 (0.17–2.40)	0.50
					A vs. T allele	0.82 (0.44–1.52)	0.53
	AA	31.6%	68.4%		TA vs. TT	1.91 (0.64–5.70)	0.24
					AA vs. TA	0.33 (0.11–1.04)	0.054

* Chi-squared test.

## Data Availability

The data that support the findings of this study are available on request from the corresponding author, A.M.

## Supplementary Materials

The following supporting information can be downloaded at: https://www.mdpi.com/article/10.3390/jcm12051963/s1, Table S1: Clinical characteristics of the follow up participants and lost in follow-up subjects. The data are presented as the mean ± SD or %. Statistically significant results are marked in bold.; Table S2: Clinical characteristics of the subjects according to progression to intermediate or late AMD stage. The data are presented as the mean ± SD or %. Statistically significant results are marked in bold.; Table S3: Differences in clinical parameters between the eyes that progressed to intermediate and to late stages of AMD. The data are presented as the mean ± SD. Statistically significant results are marked in bold.; Table S4: Differences in genetic factors between the eyes that progressed to intermediate and to late stages of AMD; Table S5: Clinical characteristics of the AMD subjects. Late-stage baseline eyes were excluded from the analysis.; Table S6: Differences in clinical features between eyes with and without progression of AMD. Eyes with baseline late AMD were excluded from analysis.; Table S7: State of disease progression with regard to the genetic background of patients. Eyes with baseline late AMD excluded from analysis.
