# Supplementary material for: Influence of Clinical and Genetic Factors on the Progression of Age-Related Macular Degeneration: A 3-Year Follow-Up
Source: J Clin Med. 2023 Mar 1;12(5):1963. doi: 10.3390/jcm12051963 (PMC10004408; doi:10.3390/jcm12051963)
Supplement: Supplementary file 1 [file jcm-12-01963-s001.zip › jcm-2169579-supplementary.pdf]

Journal of
Clinical Medicine

SUPPLEMENTARY MATERIALS

Table S1. Clinical characteristics of the follow up participants and lost in follow-up subjects. The data are presented as the mean ± SD or %. Statistically significant results are marked in bold.

Parameter		Follow up group	Lost in follow up group	p-value*
Number of subjects		94	150	—
Sex (male / female)		32/62	52/98	1.0
Patient's age [years] (min-max)		71.56 (54-85)	73.3 (49-91)	0.08
Iris colour (dark/light)		29/65	35/113	0.23
Education	Basic (%)	10.75	12.75	0.59
	Vocational (%)	15.05	16.78	
	Secondary (%)	43.01	40.94	
	Higher (%)	31.18	29.53	
AMD family history		15.96	12.84	0.57
Currently smoking		12.36	14.07	0.84
Formerly smoking		48.31	44.44	0.59
BMI (kg/m^2)		26.88 (4.53)	27.05 (3.99)	0.62
Physical activity (MET)		1585.09 (2243.62)	1755.99(2288.65)	0.26
Hypertension		67.42	62.96	0.57
History of ischemic heart disease		10.23	15.56	0.32
History of peripheral artery disease		5.68	6.67	1.0
History of limb ischemia		5.68	3.7	0.52
Hypotensive drugs/vasodilators		68.54	64.44	0.57
Thyroxine		18.18	11.85	0.24
Steroids		2.27	2.22	1.0
Statins		28.09	25.93	0.76
NSAIDs		20.22	20.0	1.0
Cardiac medications/antiarrhythmic drugs		8.99	14.81	0.22
Antiasthmatic drugs		4.49	8.15	0.41
Antidepressants		4.49	4.48	1.0
Vitamins and antioxidants		65.17	42.22	**0.001**
Xanthines (lutein, zeaxanthin)		67.42	54.81	0.07
Omega-3 rich oils		58.43	41.04	**0.013**
Resveratrol		47.19	34.81	0.07

* Mann–Whitney/Chi-squared or Fisher's exact test.

Table S2. Clinical characteristics of the subjects according to progression to intermediate or late AMD stage. The data are presented as the mean ± SD or %. Statistically significant results are marked in bold.

Parameter		Progression to intermediate stadium	Progression to late stadium	p-value*
Number of subjects		13	35	
Patient's age [years] (min-max)		70.7 (56-83)	74 (58-84)	0.31
Iris color (dark/light)[%]		30.8/69.2	20/80	0.46
Education	Basic (%)	23.1	5.7	0.08
	Vocational (%)	15.4	14.3	
	Secondary (%)	46.2	42.9	
	Higher (%)	15.4	37.1	
AMD family history (%)		7.7	17.1	0.46
Currently smoking		7.7	9.4	1.0
Formerly smoking		23.1	46.9	0.19
BMI (kg/m^2)		27.63 (5.15)	26.41 (3.82)	0.56
Physical activity (MET)		2534.42(3079)	1254(1406.6)	0.1
**Medical history**				
Hypertension		69.2	65.6	1.0
History of ischemic heart disease		15.4	9.4	0.62
History of peripheral artery disease		7.7	6.25	1.0
History of limb ischemia		7..7	6.25	1.0
**Medications use**				
Hypotensive drugs/vasodilators		69.2	68.8	1.0
Thyroxine		30.8	25.8	0.73
Steroids		0	3.1	1.0
Statins		30.8	25	0.72
NSAIDs		23.1	22.9	1.0
Cardiac medications/antiarrhythmic drugs		23.1	3.1	0.07
Antiasthmatic drugs		0	9.4	0.55
Antidepressants		0	9.4	0.55
Vitamins and antioxidants		53.9	68.8	0.5
Xanthines (lutein, zeaxanthin)		38.5	84.4	**0.004**
Omega-3 rich oils		30.8	71.9	**0.02**
Resveratrol		23.1	62.5	**0.02**

* Mann–Whitney/Chi-squared or Fisher's exact test.

Table S3. Differences in clinical parameters between the eyes that progressed to intermediate and to late stages of AMD. The data are presented as the mean ±SD. Statistically significant results are marked in bold.

Clinical parameter	Progression to intermediate stadium	Progression to late stadium	p-value*
Visual acuity (logMAR)	0.42 (0.39)	0.51 (0.34)	0.34
Choroidal thickness in the foveal region (μm)	238.23 (86.5)	196.07 (81.8)	0.11
Pachychoroid (Y/N)%	7.69/92.31	8.57/91.43	1.0
Pachyvessels (Y/N) %	38.5/61.54	40/60	1.0
Retinal thickness in the central ETDRS area (μm)	332.04 (96.6)	311.95 (75)	0.52
Soft drusen	61.54/38.46	65.71/ 34.29	1.0
Hard drusen	38.46/61.54	20/80	0.26
Subretinal drusenoid deposits (SDD)	8.33/ 91.67	54.29/45.7`	**0.007**
Pachydrusen	23.08/76.92	5.71/94.29	0.12

* Mann–Whitney/Fisher's exact test.

Table S4. Differences in genetic factors between the eyes that progressed to intermediate and to late stages of AMD.

Tested SNP	Genotype	% of patients with progression to intermediate stage	% of patients with progression to late stage	p-value*	Genotypes or alleles	OR (95% CI)	p-value*
CFH Y402H	TT	48.86	57.14	0.29	CC+TC vs TT	1.91(0.36-10.32)	0.44
					CC vs TC+TT	2.32 (0.71-14.61)	0.12
	TC	40.0	60.0		CC vs TT	3.5(0.5-24.56)	0.2
					C vs T allele	2.18 (0.81-5.82)	0.12
	CC	17.65	82.35		TC vs TT	1.13 (0.62-15.71)	0.9
					CC vs TC	3.11 (0.62-15.71)	0.16
ARMS2 A69S	GG	40.0	60.0	0.44	TT+GT vs GG	2.0 (0.5-7.0)	0.32
					TT vs GT+GG	0.63 (0.09-4.33)	0.63
	GT	21.05	78.95		TT vs GG	1.0 (0.13-7.89)	1.0
					T vs G allele	1.27(0.46-3.49)	0.64
	TT	60.0	60.0		GT vs GG	2.5 (0.55-11.33)	0.23
					TT vs GT	0.4 (0.05-3.27)	0.38
PRPH2 c.582-67T>A (rs3818086)	TT	12.5	87.5	0.32	AA+TA vs TT	0.26 (0.03-2.39)	0.32
					AA vs TA+TT	0.38 (0.06-2.21)	0.27
	TA	32.0	68.0		AA vs TT	0.14 (0.01-2.0)	0.12
					A vs T allele	0.53 (0.2-1.4)	0.2
	AA	50.0	50.0		TA vs TT	0.3 (0.03-2.9)	0.28
					AA vs TA	0.47 (0.08-2.87)	0.41

* Chi-squared test.

Table S5. Clinical characteristics of the AMD subjects. Late-stage baseline eyes were excluded from the analysis. The data are presented as the mean ± SD or %.

Parameter		AMD progression	No AMD progression	p-value*
Number of subjects		27	24	-
Patient's age [years] (min-max)		70.04 (56-81)	69.78 (54-83)	0.77
Iris colour (dark/light)		20.83/79.17	44.44/55.56	0.14
Education	Basic (%)	4.17	11.11	0.75
	Vocational (%)	20.83	14.81	
	Secondary (%)	45.83	48.15	
	Higher (%)	29.17	25.93	
AMD family history		25.0	11.11	0.28
Currently smoking		4.35	15.38	0.35
Formerly smoking		43.48	53.85	0.57
BMI (kg/m^2)		26.84 (4.5)	26.34 (4.6)	0.54
Physical activity (MET)		1609.11 (1504.3)	2079. 94 (3104.6)	0.86
Hypertension		61.54	65.22	1.0
History of ischemic heart disease		12.0	8.7	1.0
History of peripheral artery disease		0	8.7	0.22
History of limb ischemia		0	8.7	0.22
Hypotensive drugs/vasodilators		65.22	57.69	0.77
Thyroxine		69.23	30.77	0.1
Steroids		3.85	0	1.0
Statins		30.43	30.77	1.0
NSAIDs		26.09	15.38	0.48
Cardiac medications/ antiarrhythmic drugs		8.7	11.54	1.0
Antiasthmatic drugs		4.35	0	0.5
Antidepressants		4.35	0	0.5
Vitamins and antioxidants		73.91	69.23	0.76
Xanthines (lutein, zeaxanthin)		69.57	57.69	0.55
Omega-3 rich oils		60.87	53.85	0.77
Resveratrol		52.17	38.46	0.4

* Mann–Whitney/Chi-squared or Fisher's exact test.

Table S6. Differences in clinical features between eyes with and without progression of AMD. Eyes with baseline late AMD were excluded from analysis. The data are presented as the mean ± SD or %.

Clinical parameter		AMD progression	No AMD progression	p-value*
Visual acuity (logMAR)		0.39 (0.31)	0.24 (0.18)	0.24
Choroidal thickness in the foveal region (μm)		217.6 (83.05)	258 (93.93)	0.08
Pachychoroid (%)		12.50/	3.85	0.34
Pachyvessels (%)		46.15	37.50	0.58
Retinal thickness in the central ETDRS area (μm)		269.67 (25.62)	272.7 (25.6)	0.82
Soft drusen (%)		58.33	69.23	0.56
Hard drusen (%)		45.83	19.23	0.07
Subretinal drusenoid deposits (SDD) (%)		25.0	14.81	0.49
Pachydrusen (%)		12.5	11.54	1.0
AMD stage	Early (%)	29.17	14.81	0.2
	Intermediate (%)	70.83	85.19	

* Mann–Whitney/Fisher's exact test.

Table S7. State of disease progression with regard to the genetic background of patients. Eyes with baseline late AMD excluded from analysis. Statistically significant results are marked in bold.

Tested SNP	Genotype	% of patients with AMD progression	% of patients without AMD progression	p-value*	Genotypes or alleles	OR (95% CI)	p-value*
CFH Y402H	TT	37.50	62.50	0.8	CC+TC vs TT	1.4 (029-6.8)	0.67
					CC vs TC+TT	1.5 (0.44-5.09)	0.52
	TC	41.18	58.82		CC vs TT	1.67 (0.3-9.16)	0.56
					C vs T allele	1.37 (0.57-3.32)	0.48
	CC	50.0	50.0		TC vs TT	1.17 (0.21-6.56)	0.86
					CC vs TC	1.43 (0.38-5.44)	0.6
ARMS2 A69S	GG	40.0	60.0	0.69	TT+GT vs GG	1.27 (0.38-4.22)	0.7
					TT vs GT+GG	1.82 (0.24-33.7)	0.4
	GT	42.86	57.14		TT vs GG	3.0 (0.23-38.87)	0.39
					T vs G allele	1.34 (0.54-3.33)	0.53
	TT	66.67	33.33		GT vs GG	1.13 (0.32-3.9)	0.85
					TT vs GT	2.67 (0.21-34.2)	0.44
PRPH2 c.582-67T>A (rs3818086)	TT	27.27	72.73	0.38	AA+TA vs TT	2.51(0.56-11.16)	0.23
					AA vs TA+TT	0.84 (0.2-3.55)	0.82
	TA	52.17	47.83		AA vs TT	1.78 (0.28-11.12)	0.54
					A vs T allele	1.3 (0.56-3.04)	0.17
	AA	40.0	60.0		TA vs TT	2.91 (0.61-13.83)	0.17
					AA vs TA	0.61 (0.14-2.76)	0.52

* Chi-square test.
